# From Inflammation to Cutaneous Repair: Topical Application of Lupeol Improves Skin Wound Healing in Rats by Modulating the Cytokine Levels, NF-κB, Ki-67, Growth Factor Expression, and Distribution of Collagen Fibers

**DOI:** 10.3390/ijms21144952

**Published:** 2020-07-13

**Authors:** Fernando Pereira Beserra, Lucas Fernando Sérgio Gushiken, Ana Júlia Vieira, Danilo Augusto Bérgamo, Patrícia Luísa Bérgamo, Mariana Oliveira de Souza, Carlos Alberto Hussni, Regina Kiomi Takahira, Rafael Henrique Nóbrega, Emanuel Ricardo Monteiro Martinez, Christopher John Jackson, Gabriela Lemos de Azevedo Maia, Ariane Leite Rozza, Cláudia Helena Pellizzon

**Affiliations:** 1 Department of Morphology, Institute of Biosciences, São Paulo State University (UNESP), Botucatu 18618-689, São Paulo, Brazil; lucas.gushiken@unesp.br (L.F.S.G.); anajuvieira@gmail.com (A.J.V.); danilosax.tgi@gmail.com (D.A.B.); patricia.l.bergamo@gmail.com (P.L.B.); mariana.o.souza@unesp.br (M.O.d.S.); biorhn@yahoo.com.br (R.H.N.); erm_martinez@yahoo.com.br (E.R.M.M.); ariane.rozza@unesp.br (A.L.R.); claudia.pellizzon@gmail.com (C.H.P.); 2 Department of Surgery and Veterinary Anesthesiology, School of Veterinary Medicine and Animal Science, São Paulo State University (UNESP), Botucatu 18618-689, São Paulo, Brazil; cahussni@fmvz.unesp.br; 3 Department of Clinics Veterinary, School of Veterinary Medicine and Animal Science, São Paulo State University (UNESP), Botucatu 18618-681, São Paulo, Brazil; regina.takahira@unesp.br; 4 Kolling Institute of Medical Research, The University of Sydney at Royal North Shore Hospital, St. Leonard, Sydney, NSW 2065, Australia; chris.jackson@sydney.edu.au; 5 Department of Pharmacy, Federal University of São Francisco Valley (UNIVASF), Petrolina 56304-205, Pernambuco, Brazil; gabriela.lam@gmail.com

**Keywords:** cutaneous wound healing, natural product, triterpene, lupeol, skin regeneration

## Abstract

Skin wound healing is a highly complex event that involves different mediators at the cellular and molecular level. Lupeol has been reported to possess different biological activities, such as anti-inflammatory, antioxidant, antidiabetic, and in vitro wound healing properties, which motivated us to proceed with in vivo studies. We aimed to investigate the wound healing effect of lupeol-based cream for 3, 7, and 14 days. Wound excisions were induced on the thoraco-lumbar region of rats and topically treated immediately after injury induction. Macroscopic, histopathological, and immunohistochemical analyses were performed. Cytokine levels were measured by ELISA and gene expression was evaluated by real-time RT-qPCR. Our results showed a strong wound-healing effect of lupeol-based cream after 7 and 14 days. Lupeol treatment caused a reduction in proinflammatory cytokines (TNF-a, IL-1β, and IL-6) and gene and protein NF-κB expression, and positively altered IL-10 levels, showing anti-inflammatory effects in the three treatment periods. Lupeol treatment showed involvement in the proliferative phase by stimulating the formation of new blood vessels, increasing the immunostaining of Ki-67 and gene expression, and immunolabeling of vascular endothelial growth factor (VEGF) and epidermal growth factor (EGF), and increasing gene expression of transforming growth factor beta-1 (TGF-β1) after seven days of treatment. Lupeol was also involved in the tissue regeneration phase by increasing the synthesis of collagen fibers noted in the three treatment periods analyzed. Our findings suggest that lupeol may serve as a novel therapeutic option to treat cutaneous wounds by regulating mechanisms involved in the inflammatory, proliferative, and tissue-remodeling phases.

## 1. Introduction

Wound healing is a fascinating but complicated process, affected by several factors that contribute jointly to wound closure, including blood coagulation, inflammation, fibroplasia, collagen deposition, and wound contraction [1]. The success of skin restructuring is dependent on a cascade of ordered events involving cellular, biochemical, and molecular responses and/or interactions. For didactic reason, this process is analyzed from three overlapping phases: inflammatory phase, formed by events such as hemostasis and inflammation; proliferative phase, characterized by granulation tissue formation, angiogenesis, and re-epithelialization; and the remodeling phase of the extracellular matrix [2].

Studies developed with biomolecules for the development of new drugs or new therapeutic agents represent a possibility for the treatment of different diseases and inflammatory conditions, including skin wounds [3]. Natural products are a source of compounds with potential biological activity and certain species of plants or natural substances isolated from them have been used successfully in recent studies on the treatment of cutaneous wounds [4]. Secondary metabolites or active compounds isolated from many natural sources, in addition to plants, have also been shown to be responsible for the induction of skin wound healing in animal models [5].

The natural triterpene lupeol is a bioactive found in various edible plants, such as olive, fig, mango, carrot, soybean, melon seed, and grapes, and also in medicinal plants such as *Bowdichia virgilioides* [6]. Several studies have shown pharmacological potential of lupeol, including antioxidant, anti-inflammatory, antihyperglycemic, anti-dyslipidemic, antiarthritic, cardioprotective, hepatoprotective, and wound-healing effects in experimental models in vivo and in vitro [7,8,9,10]. We have previously reported that lupeol at low concentrations was able to stimulate the proliferation, migration, and cell contraction by promoting wound healing in human keratinocytes and fibroblasts possibly through involvement of PI3K/Akt and p38/ERK/MAPK pathways [11].

A previous study has reported wound-healing activity of the lupeol-based gel applied topically on rat wounds during 16 days of treatment [12]. Based on this work, a recent study developed by our group demonstrated an important role for lupeol on hyperglycemia-induced impaired wound healing after 14 days of treatment. The results revealed a significant decrease in the inflammatory process, better formation of granulation tissue, and increased vascularization and deposition of collagen fibers after lupeol treatment [13]. However, the effectiveness of lupeol on skin wound healing after different treatment periods has not been investigated, and its underlying mechanisms in normoglycemic rats remain unknown. In the present study, we examined the effects of treatment with lupeol-based cream on wounds of normoglycemic rats after 3, 7, and 14 days of treatment, in addition to determining the involvement of the main mediators involved.

## 2. Results

### 2.1. Macroscopic Analysis of Lupeol Treatment in Cutaneous Wounds

**Figure 1:**
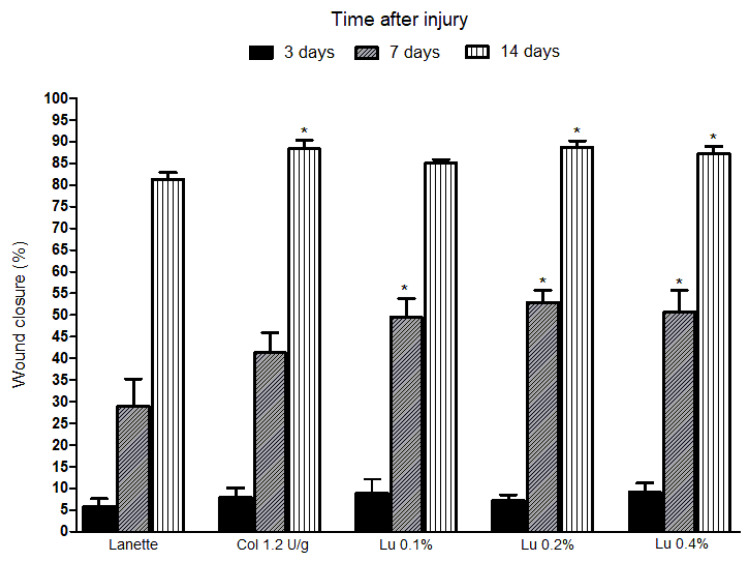
Effect of lupeol on the healing of excisional wounds in rats. Wound closure percent of the animals treated with Lanette, collagenase 1.2 U/g, or lupeol 0.1%, 0.2%, or 0.4% after 3, 7, and 14 days. * *p* < 0.05 vs. Lanette group by ANOVA followed by the Newman–Keuls test.

The wound reduction was calculated after 3, 7, and 14 days of lesion induction in rats. Wound closure was notably improved after 7 and 14 days of lupeol treatment. Initially, the lesions were analyzed throughout the three days of treatment, during which no significant changes were observed in wound closure rate between the treatments. After 7 days of treatment, it was possible to observe a statistically significant retraction of the lesions in the lupeol-treated groups at all concentrations studied in relation to the Lanette group. The lesions analyzed after 14 days of treatments with lupeol at 0.2% and 0.4% showed a significant 89% and 87% increase, respectively, in the wound reduction rate compared to the Lanette group. The collagenase (reference drug) group showed an 88% significant increase in wound reduction only after 14 days of treatment in relation to the Lanette group, and when compared to lupeol-treated groups (in all concentrations), no significant differences were observed in the wound closure percentage, as can be seen in Figure 1.

We also evaluated the clinical parameters such as exudation, edema, hemorrhage, presence of crust, and granulation in all rats (Table 1). After three days of treatment, we observed a significant reduction in the exudation process of the groups treated with collagenase, and lupeol 0.2% and 0.4% in relation to the Lanette group. After seven days, both groups treated with lupeol also demonstrated a significant increase in the granulation process compared to Lanette, but only the higher dose (0.4%) significantly increased crust formation compared to Lanette treatment. The lesions analyzed after 14 days of treatment showed that the Lanette-treated animals still exhibited a small amount of crust adhered to the lesion, whereas groups treated with lupeol 0.2% and 0.4% did not possess this crust, showing only the scar of the injured region. There were no significant changes in edema and hemorrhage parameters analyzed after three and seven days of wound induction. According to the results of the macroscopic analysis, we selected the 0.2% concentration as the lowest effective concentration to determine histopathological, immunoenzymatic, and molecular changes.

### 2.2. Histological Examination

**Figure 2:**
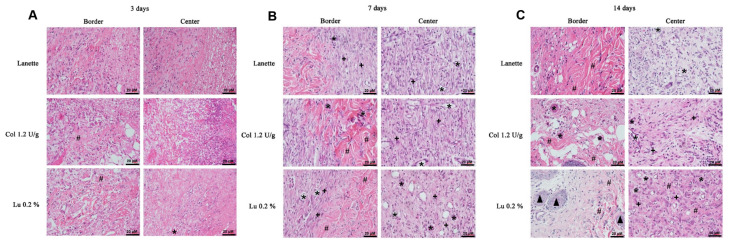
HE-stained skin tissue sections on Day 3 (**A**), 7 (**B**), and 14 (**C**) post wound induction at high magnification. Bar represents 20 μm. (**#**) indicates presence of collagen fibers, (*) blood vessels, (**+**) fibroblasts, and (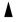) sebaceous glands.

**Figure 3:**
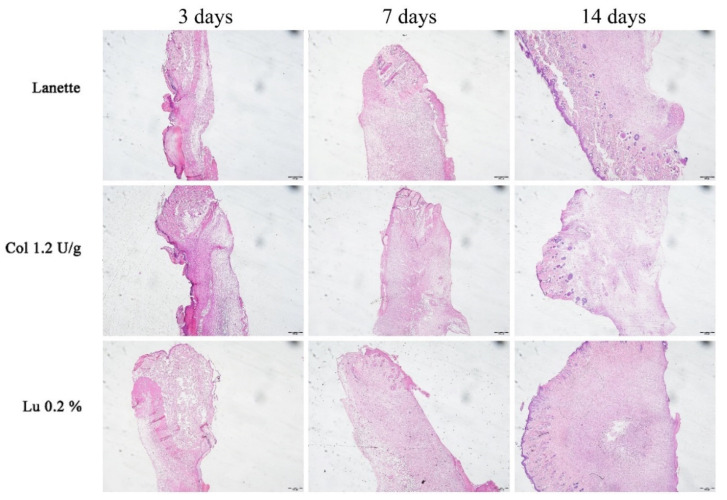
HE-stained skin tissue sections on Day 3, 7, and 14 post wound induction at low magnification. Bar represents 500 μm.

**Figure 4:**
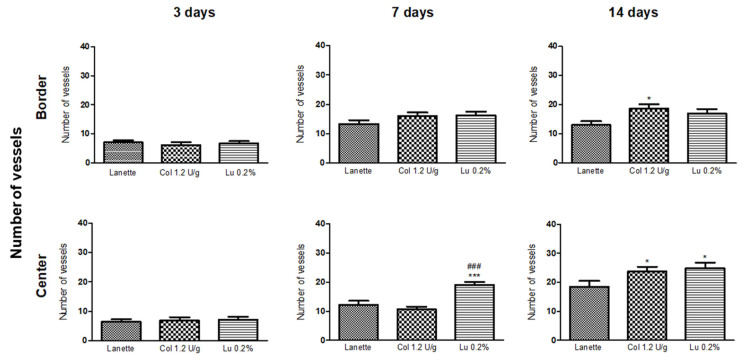
Number of blood vessels in HE staining of the border and central region of rat cutaneous lesions treated with Lanette, collagenase 1.2 U/g, or lupeol 0.2% for 3, 7, and 14 days. * *p* < 0.05 and *** *p* < 0.001 vs. Lanette group. ### *p* < 0.001 vs. collagenase group, using ANOVA followed by the Newman–Keuls test.

The HE staining allowed the counting of blood vessels in the border and the lesion center, as you can see in Figure 2A–C. An overview of skin tissue sections on Day 3, 7, and 14 post wound induction in low magnification is shown in Figure 3. It was not possible to observe significant change in vascularization of these regions after three days of lesion induction. However, we observed a significant increase in the number of blood vessels in the central region of the lesion in the lupeol-treated groups compared to the Lanette- and collagenase-treated groups. After 14 days of treatment, there was a significant increase in vascularization in the border region in the collagenase treatment compared to Lanette. There were significant vascular alterations in the wound center in the groups treated with collagenase and lupeol compared to the Lanette group (Figure 4).

**Figure 5:**
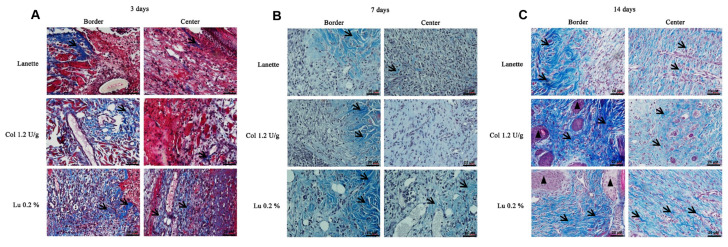
Masson’s trichrome-stained skin tissue sections on Day 3 (**A**), 7 (**B**), and 14 (**C**) post wound induction at high magnification. Bar represents 20 μm. (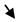) indicates the presence of total collagen fibers and (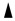) sebaceous glands.

**Figure 6:**
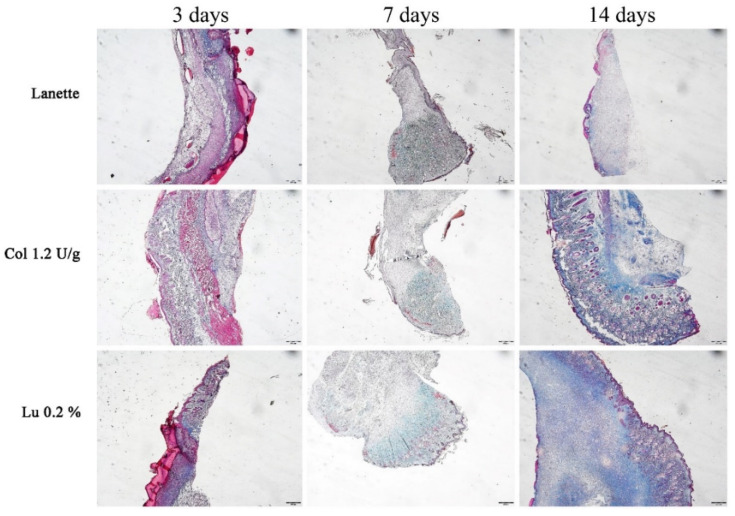
Masson’s trichrome-stained skin tissue sections on Day 3, 7, and 14 post wound induction at low magnification. Bar represents 500 μm.

**Figure 7:**
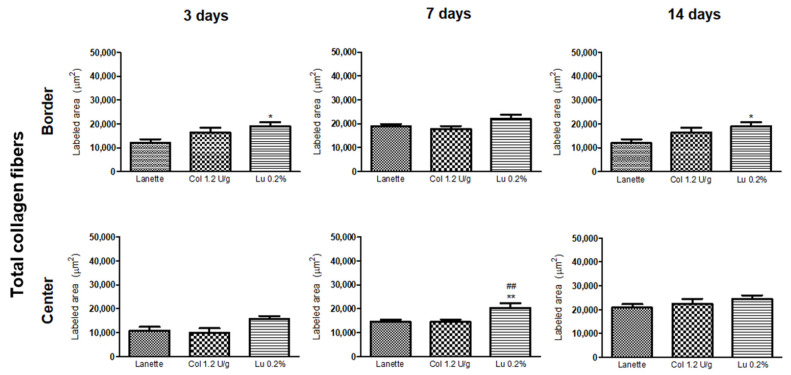
Labeling area of total collagen fibers (μm^2^) in the border and central region of rat cutaneous lesions treated with Lanette, collagenase 1.2 U/g, or lupeol 0.2% for 3, 7, and 14 days. * *p* < 0.05 and ** *p* < 0.01 vs. Lanette group. ## *p* < 0.01 vs. collagenase group, using ANOVA followed by the Newman–Keuls test.

Analysis of total collagen fibers using Masson’s trichrome method showed labeled area (μm^2^) in the border and center regions of the lesion (Figure 5A–C). An overview of skin tissue sections on Day 3, 7, and 14 post wound induction at low magnification is shown in Figure 6. There was a significant increase in the labeling of these proteins in the border region of the lesion of the lupeol-treated group in relation to the Lanette group, after three days of treatment. On Day 7, we observed a significant increase in the deposition of total collagen fibers in the wound center of the lupeol treatment in relation to the Lanette- and collagenase-treated groups. By Day 14, only the lupeol-treated group showed a significant increase in the labeled area by total collagen fibers at the border of the lesions compared to Lanette (Figure 7).

### 2.3. Effect of Lupeol on the Immunostaining of NF-κB, Ki-67, EGF, and VEGF

**Figure 8:**
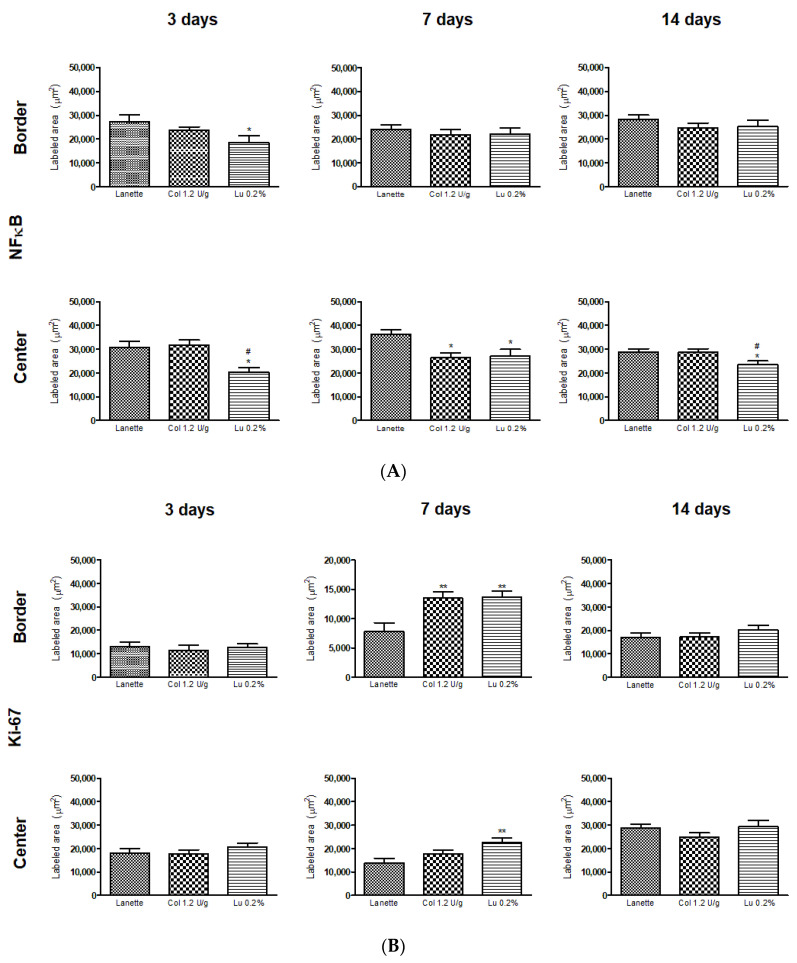
Immunolabeled area (μm^2^) for NF-κB (**A**) and Ki-67 (**B**) in the border and central region of rat cutaneous lesions treated with Lanette, collagenase 1.2 U/g, or lupeol 0.2% for 3, 7, and 14 days. * *p* < 0.05 and ** *p* < 0.01 vs. Lanette group. # *p* < 0.05 vs. collagenase group, using ANOVA followed by the Newman–Keuls test.

**Figure 9:**
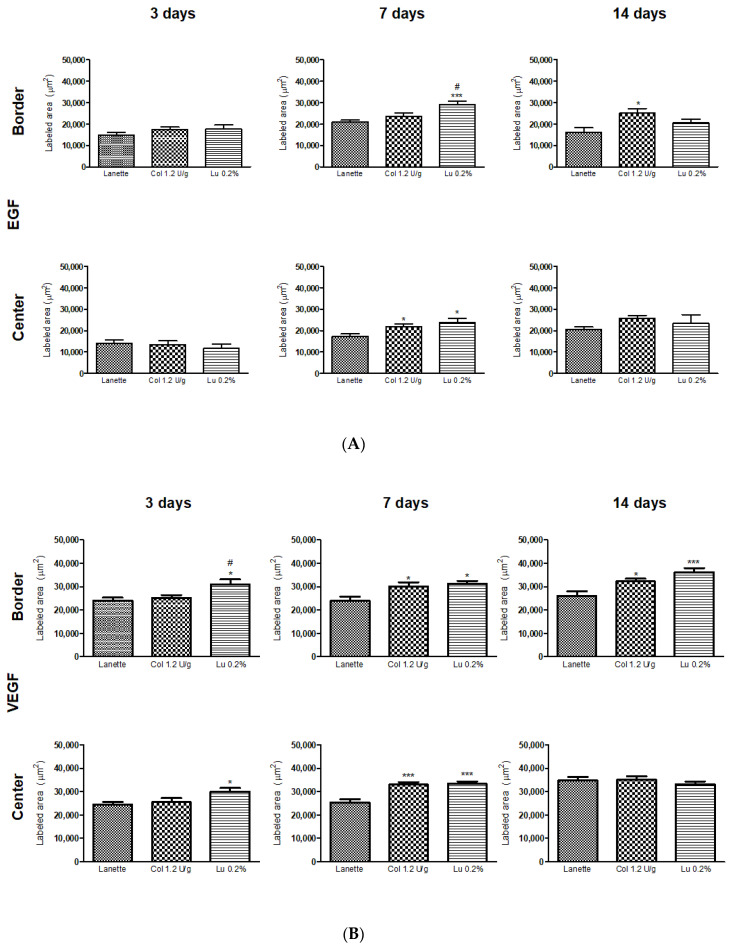
Immunolabeled area (μm^2^) for EGF (**A**) and VEGF (**B**) in the border and central region of rat cutaneous lesions treated with Lanette, collagenase 1.2 U/g, or lupeol 0.2% for 3, 7, and 14 days. * *p* < 0.05 and *** *p* < 0.001 vs. Lanette group. # *p* < 0.05 vs. collagenase group, using ANOVA followed by the Newman–Keuls test.

Immunohistochemical analysis for NF-κB after three days of treatment showed a significant decrease in the border and lesion center immunolabeling of the lupeol-treated group compared to Lanette and collagenase treatments. After seven days of treatment, a significant decrease in NF-κB was observed only at the wound center of collagenase- and lupeol-treated animals compared to the Lanette group. On Day 14, the lupeol-treated group showed a significant decrease in NF-κB compared to the Lanette or collagenase groups (Figure 8A). Immunolabeling to localize Ki-67 performed after seven days of treatment showed a significant increase in the antibody-labeled area in the border and lesion center of the lupeol-treated group compared to Lanette treatment. Analysis carried out after 3 and 14 days of treatment did not show significant changes in the groups tested (Figure 8B). Regarding EGF, after three days, there was no significant change in any groups tested (Figure 9A), however, by Day 7, there was a significant increase in staining of the border area for the lupeol treatment compared to control treatment. In the lesion center, both lupeol and collagenase treatments significantly increased the EGF-immunolabeled area in relation to the Lanette treatment. After 14 days, only collagenase-treated rats showed a significant increase in the wound border compared to the Lanette treatment. The immunohistochemistry of VEGF showed that the lupeol treatment significantly increased the immunolabeling of the lesion border compared to the Lanette group after three and seven days of treatment. Collagenase treatment also showed a significant increase of the immunolabeled area in the borders compared to the Lanette group after seven days of experimentation. After 14 days, a significant increase of the immunolabeling was observed in the lupeol- and collagenase-treated groups compared to Lanette. Lupeol treatment also caused a significant increase of the immunolabeled area in the lesion center after three and seven days of treatment compared to Lanette, while the collagenase treatment increased VEGF immunolabeling in the lesion center only after seven days of treatment (Figure 9B). The photomicrographs of the immunohistochemical analyses can be found in the Figures S1–S8.

### 2.4. Effect of Lupeol on Pro- and Anti-Inflammatory Cytokine Levels

**Figure 10:**
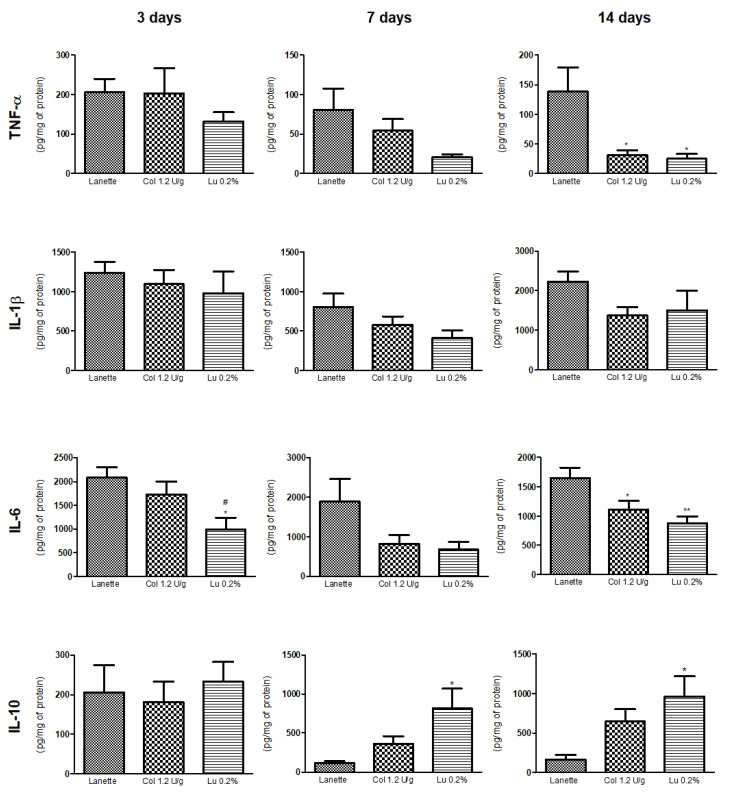
Quantification of TNF-α, IL-1β, IL-6, and IL-10 levels (pg/mg protein) in rat cutaneous lesions treated with Lanette, collagenase 1.2 U/g, or lupeol 0.2% for 3, 7, or 14 days. * *p* < 0.05 and ** *p* < 0.01 vs. Lanette group. # *p* < 0.05 vs. collagenase group, using ANOVA followed by Newman–Keuls test.

ELISA results showed an anti-inflammatory effect of lupeol through a significant reduction of TNF-α levels as compared to the Lanette group after 14 days of treatment. On Days 3 and 14, the lupeol-treated group caused a significant decrease in IL-6 levels as compared to Lanette treatment. There was also a significant increase in IL-10 levels in lupeol-treated rats compared to the Lanette group after 7 and 14 days of treatment. Lupeol did not cause any significant changes in IL-1β levels in the three periods studied (Figure 10).

### 2.5. Effect of Lupeol on Nf-κb, Ki-67, Egf, Vegf-A, and Tgf-β1 mRNA Expression

**Figure 11:**
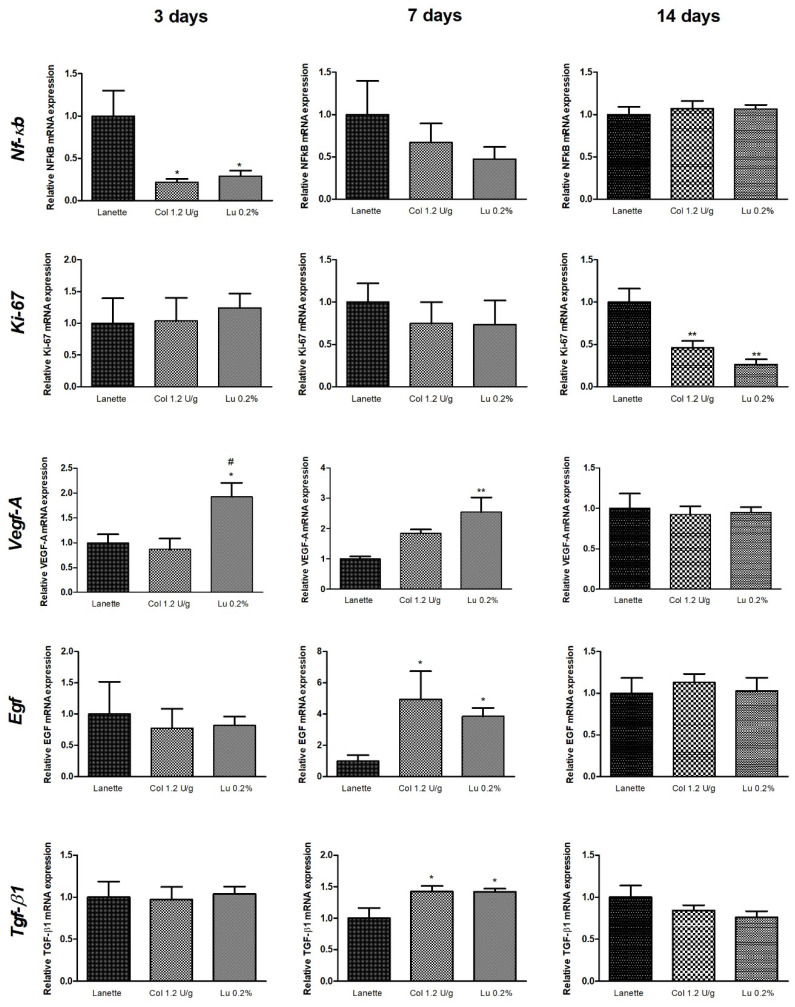
Gene expression (RT-qPCR) of *Nf-kb*, *Ki-67*, *Vegf-A*, *Egf*, and *Tgf-β1* in rat cutaneous lesions treated with Lanette, collagenase 1.2 U/g, or lupeol 0.2% for 3, 7, or 14 days. * *p* < 0.05 and ** *p* < 0.01 vs. Lanette group. # *p* < 0.05 vs. collagenase group, using ANOVA followed by Newman–Keuls test.

The molecular analyses of the real-time gene expression of *Nf-κb*, *Ki-67*, *Egf*, *Vegf-A,* and *Tgf-β1* are shown in Figure 11. There was a decrease in *Nf-κb* expression in the collagenase- and lupeol-treated groups compared to Lanette after three days. The relative expression of *Ki-67* decreased significantly in the collagenase- and lupeol-treated groups compared to Lanette after 14 days of treatment. Lupeol treatment caused a significant increase in the expression of *Vegf-A* compared to Lanette after three and seven days of treatment. After 14 days, a reduction of the *Vegf-A* expression in the lupeol-treated rats compared to the collagenase group was observed. There was an increase in the gene expression of *Egf* and *Tgf-β1* in the collagenase- and lupeol-treated groups compared to Lanette only after seven days of treatment (Figure 11).

## 3. Discussion

The use of medicinal plants and isolated bioactive substances appears as an alternative to assist in the cicatricial process, reducing wound closure time, re-epithelialization, and tissue fibrosis, due to the presence of secondary metabolites such as tannins, steroids, terpenes, flavonoids, alkaloids, coumarins, and saponins that act in the different stages of cutaneous wound healing through various mechanisms of action involved [5]. The present study confirms the efficacy of lupeol isolated from *B. virgilioides* as analyzed by macroscopic, histopathological, biochemical, and molecular parameters, showing an anti-inflammatory effect and a significant increase in pro-angiogenic and re-epithelialization markers, and of stimuli in the deposition of collagen fibers in dermal wounds treated after 3, 7, and 14 days in vivo.

In general, natural products have been considered an important source of bioactive molecules with therapeutic potential in cutaneous wound treatment [14]. Proteolytic enzymes have been used as a debriding agent in the treatment of these injuries [15]. Collagenase, an enzyme debriding agent derived from *Clostridium histolyticum*, is used in the clinic for the treatment of infected and surgical wounds [16]. Previous studies have reported that collagenase acts by cleaning the necrotic tissue of the wound through an enzymatic method, accelerating the formation of granulation tissue and subsequent re-epithelialization, and increasing the activation of fibroblasts, myofibroblasts, and collagen in dermal lesions of rats [17]. Therefore, collagenase was used in our study as a positive control for treatment of rat skin wounds.

The mechanism of retraction of cutaneous wounds involves a series of interactions between cells and extracellular matrix molecules during the three healing phases, capable of generating mechanical forces that result in the wound closure [18]. The inflammatory phase of the healing process is essential for correct tissue repair of the lesions. Inflammatory cells such as neutrophils and activated macrophages trigger the release of inflammatory mediators such as cytokines, chemokines, reactive oxygen species, and proteolytic enzymes. The production of inflammatory mediators is finely regulated and one of the key regulatory points of these is the inhibition of transcription factors, such as factor nuclear kappa B (NF-κB). This protein complex is formed by cytoplasmic subunits that are inactive, and when activated, they translocate to the nucleus and bind to the consensus region of genes that express cytokines and oxidizing enzymes. Because of this, NF-κB has been considered an important inflammatory marker and a target molecule for the treatment of inflammatory disorders [19]. Our results showed a strong reduction in the gene expression of NF-κB after three days of treatment and in the immunolabeling of this antibody in the three periods studied. These results corroborate with recent findings in vitro and in vivo, where lupeol was able to suppress the activation of NF-κB in keratinocytes and in streptozotocin-induced hyperglycemic rat wounds, respectively [11,13].

TNF-α is a proinflammatory cytokine, which, during the inflammatory phase of the cicatricial process of lesions, is expressed in greater quantity when compared to its normal levels. IL-1β is proinflammatory and synthesized by different cell types, such as monocytes and macrophages, as well as other endothelial cells, and among the main functions of IL-1β is the contribution with local inflammatory processes in the injury and promotion of the secretion of other proinflammatory cytokines such as IL-6 and TNF-α [20]. IL-6 also acts as a multifunctional cytokine with pleiotropic activities in inflammation, immune responses, and hematopoiesis [21]. Together with TNF-α and IL-1β, IL-6 is present in high concentrations in a wide variety of disease states associated with inflammation [22]. Another cytokine involved in healing is anti-inflammatory interleukin (IL-10), which is related to the inhibition of the synthesis of proinflammatory cytokines such as TNF-α, IL-1β, and IL-6 in active macrophages in the inflammatory process [23], and is also involved in the angiogenesis process [24]. Inhibition of exacerbated expression of proinflammatory cytokines and increased expression of anti-inflammatory cytokines are involved as an essential healing mechanism [25]. Some studies have demonstrated that lupeol is associated with the modulation of various inflammatory agents, such as TNF-α, IL-1β, and IL-6 [26]. Recently, we have shown that lupeol treatment of hyperglycemic rat wounds was able to favorably regulate cytokine levels, to provide a net anti-inflammatory effect [13]. The present study confirms this potential anti-inflammatory effect of lupeol in cutaneous lesions of rats by significantly reducing the levels of proinflammatory cytokines such as TNF-α and IL-6 after 3 and 14 days of treatment and increasing the IL-10 levels after 7 and 14 days of treatment.

The formation of granulation tissue, constituted by macrophages, fibroblasts, and neoformed vessels, is fundamental for the re-epithelialization process and reconstructing of the extracellular matrix [27]. Increased cell proliferation is a crucial aspect of wound healing in general, and the Ki-67 protein is an important marker of this cellular event. Ki-67 expression is also widely known to be an indicator of cell growth within a total cell population [28]. Our results showed a reduction in Ki-67 gene expression in the lupeol-treated group after 14 days. In contrast, we also observed increased expression of this protein in the immunohistochemical analysis for the lupeol treatment after seven days of treatment, suggesting that lupeol increases cell proliferation and the consequent stimulus for the next phase of the cicatricial process, the remodeling phase.

Another essential event in wound healing is re-epithelialization, which begins a few hours after injury, but shows more evident activity in the proliferative phase and can continue until the extracellular matrix remodeling phase [27]. Epidermal growth factor (EGF) is a crucial marker of this phase and possesses mitogenic and migratory activity on the edge keratinocytes [29]. Our results showed increased gene expression of EGF in the lupeol-based cream treatment, as well as increased immunostaining for this growth factor after 7 and 14 of treatments analyzed in the border and lesion center, and that way, confirming the role of the lupeol on re-epithelialization in the healing process.

The angiogenic process is an important part of the proliferation phase and involves variable growth factors such as platelet-derived growth factor (PDGF), fibroblast growth factor-2 (FGF-2), transforming growth factor-beta 1 (TGF-β1), vascular endothelial growth factor (VEGF), and angiotensin [30]. VEGF, in particular, is one of the most potent mediators of angiogenesis, considered to be a pivotal process in wound healing, contributing to the formation of new vessels by stimulating the survival, proliferation, and migration of endothelial cells. This event is fundamental because it promotes nutrition through the supply of oxygen and other essential substances in tissue repair [31,32]. The data obtained in our study showed an increase in the number of blood vessels in the border and lesion center in the lupeol-treated groups after 7 and 14 days, together with an increase in gene expression observed after 3 and 7 days of treatment and in the immunolabeling of VEGF during the three treatment periods tested. The increased number of blood vessels associated with upregulation of VEGF clearly demonstrates the role of lupeol on angiogenesis for cutaneous wound healing.

TGF-β1 is reported to be one of the proteins with the broadest spectrum of activities, which displays effects on the inflammatory process, cell differentiation and extracellular matrix production, evolution of granulation tissue through the recruitment of inflammatory leukocytes, and stimulation of fibroblasts and epithelial cells [33]. Our results showed an increase in the gene expression of TGF-β1 in the lupeol-treated group after seven days corresponding to the proliferative phase, which mediates the formation of granulation tissue and local neovascularization. These data corroborate our previous study, where there was an increase in the expression of TGF-β in the lupeol-treated group in diabetic rat wounds [13].

Finally, the regeneration phase, which involves extensive tissue remodeling, is replaced by proteoglycan and collagen molecules, which organize in thicker bundles, resulting in intact and resistant tissue [34]. It is interesting to emphasize that synthesis of collagen fibers is one of the most important events in the wound-healing process [35]. Our study showed an increase in the deposition of collagen fibers after 3, 7, and 14 days of treatment with lupeol, indicating that lupeol treatment is also effective in the tissue regeneration phase by increasing the synthesis of collagen fibers.

## 4. Materials and Methods

### 4.1. Plant Material and Extraction and Isolation of Lupeol

Stem bark of *Bowdichia virgilioides* Kunth. was collected in December 2014 in a coastal area of the Atlantic Forest, in the surroundings of Santa Rita, Paraíba, Brazil. The sample was identified by comparison with the plant specimens and deposited in the Herbarium Prof. Lauro Pires Xavier and in the reference collection of the Laboratory of Pharmaceutical Technology from Federal University of Paraíba, João Pessoa, Brazil, under number Agra et Góis 6243. The plant was dried (50 °C) and then powdered. The powder (3.0 kg) was extracted with 95% ethanol, filtered, and concentrated under vacuum to obtain the crude ethanolic extract (EtOHE, 250 g). Afterwards, the EtOHE was suspended in a MeOH/H_2_O mixture (2:3) and subjected to the liquid–liquid partitioning process with hexane. The hexane residue (49 g) was subjected to repeated washings with acetone under stirring, followed by filtration. The solid obtained was recrystallized from chloroform and hexane, resulting in white crystals which were examined by analyzing ^1^H and ^13^C NMR spectral data, compared with those published in the literature [36], and identified as lupeol [11] substance (3 g).

### 4.2. Animals

Healthy male *Wistar* rats (*Rattus norvegicus*) were procured from Central Animal House, UNESP, Botucatu. They weighed between 180 g and 220 g, and their average age was 8 weeks. The animals were housed individually in polyethylene cages in an experimental animal room with a 12 h light/dark cycle. They were maintained at a room temperature of 23 ± 2 °C and humidity at 55% ± 15%. The rats were fed a standard diet and water ad libitum, and they were acclimatized for at least one week before the experiment. All experiments were carried out in accordance with the experimental protocols (Protocol 610/2014) approved (22/05/2014) by the Ethics Committee on Animal Use (CEUA/IBB/UNESP) [37].

### 4.3. Excision Wound Model

All rats were anesthetized with ketamine hydrochloride (0.08 mg/100 g) and xylazine (0.04 mg/100 g) by intraperitoneal injection. Prior to the surgical procedure, the animals received a single dose of ketoprofen (100 mg/kg, SC) as an ethical conduct to minimize postoperative discomforts. After shaving the hair on the back of each rat, the skin was sterilized with 70% alcohol to remove any type of contamination and a lesion was created in the posterior dorsal region of each animal using a 2 cm diameter punch. Thereafter, animals were housed individually and monitored in properly disinfected cages to prevent infection or further damage to the wounds after recovering from anesthesia [13].

### 4.4. Grouping and Topical Treatment

We determined the choice of concentrations in this work based on a previous study published by Harish et al. (2008) [12], in which he showed wound healing activity of lupeol-based gel at 0.2%. Here, we have defined the same concentration and included two more in this study, 0.1% and 0.4%. We also used the Lanette cream as a vehicle for the formulation, which causes greater stability to substances that can be incorporated in both the aqueous and oily phases, and is able to be used in a larger area without the risk of rapid evaporation [38].

To assess the cutaneous wound healing of the lupeol-based cream, wound excision models were used. The rats were divided into five groups (n = 8), including three doses of lupeol cream (0.1, 0.2, and 0.4% *w*/*w*), as shown below:

- Group I—Topically treated with Lanette cream (vehicle)
- Group II—Topically treated with collagenase 1.2 U/g (reference drug)
- Group III—Topically treated with 0.1% *w*/*w* lupeol cream (substance test)
- Group IV—Topically treated with 0.2% *w*/*w* lupeol cream (substance test)
- Group V—Topically treated with 0.4% *w*/*w* lupeol cream (substance test)

Immediately after the surgical excision, the wounds were topically treated for three different experimental periods: 3, 7, or 14 days, according to the stages described in the literature: inflammatory, proliferative, and remodeling [27,39]. Formulations were applied topically every day, once a day during each period. The rats were placed in their respective cages with one rat per cage.

### 4.5. Determination of Wound Retraction Percentage

The wound closure was analyzed daily in each treatment period group using transparency paper and a permanent marker. After scanning, the wound area was measured using specific software Adobe Photoshop C5-version 5 (Adobe Systems Inc, San Jose, CA, USA). The area of wound retraction was calculated (%) by the following formula: % wound retraction = {(initial area of the wound − area of wound measured)/initial area of the wound} × 100. The data of the wound areas were expressed as the mean ± standard deviation [39].

### 4.6. Macroscopic Examination

The clinical signs of the lesions, such as exudation, edema, local hemorrhage, presence of crust and granulation tissue, were monitored by macroscopic examination and graded on a four-point scale: 0—absent (0%), 1—light (30%), 2—moderate (30–70%), and 3—intense (>70%) based on the method from Oliveira et al. (2014) [40], with modifications.

### 4.7. Histological Analysis

**Figure 12:**
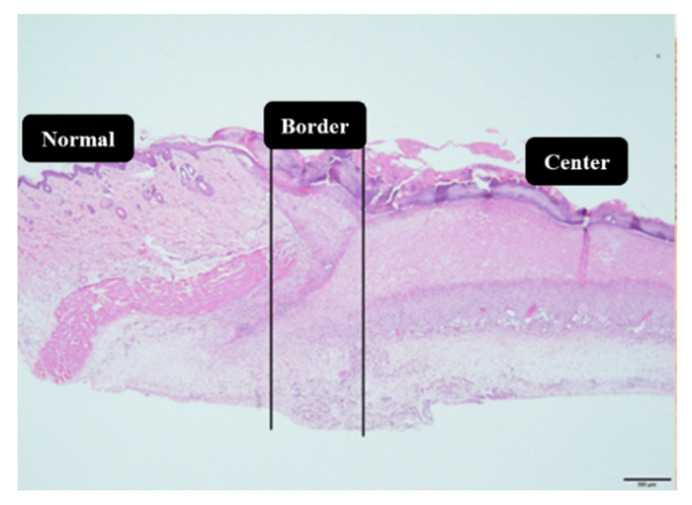
Overview of the wound areas (normal, border, and center of the lesion) analyzed in the histological and immunohistochemical sections. Scare bar: 500 μM.

Skin samples were fixed with alcohol, formalin, and acetic acid (8:1:1) and processed in paraffin. The number of blood vessels and deposition of collagen fibers were assessed by hematoxylin and eosin (HE) and Masson’s trichrome staining, respectively. For all analyses (histological and immunohistochemistry), three distinct regions were photographed: normal skin (skin without wound), border, and the center of the wound, as can be seen in Figure 12. Ten photomicrographs of each sample were analyzed under a 40× magnification, and scored as previously described [13,39], being 5 for the border and 5 of the central lesion regions. The numbers of blood vessels and collagen fibers labeled in these regions were quantified by the marked area count, totaling an area of 100,000 μm^2^/slice. The photomicrographs were obtained with the software CellSens Standard (Olympus, Center Valley, PA, USA) and the measurements were made using AVSoftBioView software.

### 4.8. Immunohistochemistry Analysis

The immunohistochemical studies were carried out with sections stained with monoclonal antibodies against NF-κB (1:100 μL), Ki-67 (1:200 μL), EGF (1:200 μL), and VEGF (1:100 μL). The same samples used for histological analysis were cut (5 μm) and fixed on silanized slides and submitted to antigen recovery by pressure (20 psi/125 °C) and the immunohistochemical reaction was performed with a polymer kit. Ten photomicrographs (5 in the border and 5 in the central lesion regions, as shown in Figure 9) of each slice were analyzed with 40× magnification software CellSens Standard (Olympus, Center Valley, PA, USA) [13,39]. The immunolabeled area was quantified totaling 100,000 μm^2^/slice. The quantification was made with the software AVSoftBioView.

### 4.9. Enzyme-Linked Immunosorbent Assay (ELISA)

The skin fragments removed for immunoenzymatic analyses were homogenized in a mixture of cold PBS supplemented with protease inhibitor cocktail (Sigma-Aldrich, St. Louis, MO, USA) and centrifuged at 10,000 rpm for 20 min at 4 °C. Tissue homogenate was centrifuged and the supernatant was processed to determine the levels of the proinflammatory cytokines TNF-α, IL-1β, and IL-6 and the anti-inflammatory cytokine IL-10 using commercial enzyme-linked immunosorbent assay (ELISA) kits (R&D Systems, Minneapolis, MN, USA). Measurements of total proteins were determined by the biuret assay so that the data of the parameters analyzed are expressed relative to the amount of protein in the sample in milligrams [41].

### 4.10. RNA Extraction and Reverse Transcription Quantitative PCR (RT-qPCR)

A portion of the lesion samples from each animal was collected and frozen at −80 °C. The samples were weighed and 100 mg of each one was macerated using liquid nitrogen, and subsequently placed in microtubes. The RNA of the skin samples (*Rattus norvegicus*) was extracted using the TRIzol method (Invitrogen, Carlsbad, CA, USA), following the manufacturer’s recommendations. To avoid genomic DNA contamination, the RNA samples were treated with DNase I, RNase-free kit (Invitrogen, Carlsbad, CA, USA) prior to cDNA synthesis. The cDNA synthesis was performed with random hexamers using Supercript^®^ II (Invitrogen, Carlsbad, CA, USA) according to standard protocols [42]. The quantitative PCR reaction was carried out using designed and specific forward and reverse primers for *Rattus norvegicus* (Table 1) to evaluate the expression of the genes for *Nf-κb*, *Ki-67*, *Egf*, *Vegf*, and *Tgf-β1*, among the different treatments used. For qPCR, Cts values were determined using SYBR Green kit (Invitrogen, Carlsbad, CA, USA). For each gene of interest, the mRNA levels (Cts) were normalized by the reference gene, *β-actin* (Table 2), and expressed with values relative to the Cts mean (point from which the system starts the quantification of the genetic material from the exponential phase threshold) of each group (ddCt-Ct normalized by means of the respective *β-actin* groups). All qPCR reactions (10 µL) used 900 nM for each primer and 700 ng of total RNA. Each reaction was performed in duplicate in a StepOne system (Life Technologies, Carlsbad, CA, USA) following the manufacturer’s instructions, and relative gene expression profiles were calculated, according to the ΔΔ*C*T method as previously described [43].

### 4.11. Statistical Analysis

Statistical analysis was performed using GraphPad Prism 5.01 software. The nonparametric data were expressed as median (maximum and minimum) and performed by the Kruskal–Wallis test, followed by the Dunn test. Parametric data were expressed as mean ± standard error of the mean and the comparison between groups was performed by ANOVA followed by the Newman–Keuls test. Values of *p* < 0.05 were considered statistically significant.

## 5. Conclusions

Based on our results, we conclude that lupeol from *B. virgilioides* accelerates cutaneous wound healing via several mechanisms, including: anti-inflammation, through the modulation of NF-κB expression and pro- and anti-inflammatory cytokines; promoting new granulation tissue, angiogenesis, and re-epithelialization, indicated by modulating Ki-67, VEGF, EGF, and TGF-β1, and stimulation of the synthesis of collagen fibers; contributing to the tissue remodeling. Our findings confirm the wound-healing potential of lupeol, suggesting that this triterpene is a promising molecule for therapeutic use.

## Figures and Tables

**Table 1:** Clinical parameters of cutaneous lesions after topical treatment with Lanette, collagenase 1.2 U/g, or lupeol 0.1, 0.2, or 0.4% (n = 8) in rats.

Parameters	Groups	Days after Wound Induction		
		3 Days	7 Days	14 Days
**Exudation**	Lanette	2 (2, 3)	1 (0, 3)	–
	Collagenase	1 (0, 2) *	0 (0, 1)	–
	Lupeol 0.1%	1 (1, 2)	0 (0, 1)	–
	Lupeol 0.2%	1 (0, 3) *	0 (0, 1)	–
	Lupeol 0.4%	1 (0, 3) *	0 (0, 1)	–
**Edema**	Lanette	2 (1, 3)	–	–
	Collagenase	1 (0, 2)	–	–
	Lupeol 0.1%	1 (1, 3)	–	–
	Lupeol 0.2%	1 (0, 2)	–	–
	Lupeol 0.4%	1 (0, 3)	–	–
**Hemorrhage**	Lanette	1 (0, 3)	1 (0, 2)	–
	Collagenase	0.5 (0, 1)	0 (0, 1)	–
	Lupeol 0.1%	1 (0, 2)	0.5 (0, 2)	–
	Lupeol 0.2%	0 (0, 2)	0 (0, 1)	–
	Lupeol 0.4%	0 (0, 1)	0 (0, 2)	–
**Presence of crust**	Lanette	0 (0, 2)	1.5 (0, 3)	1 (1, 3)
	Collagenase	1 (0, 3)	2 (1, 3)	1 (0, 3)
	Lupeol 0.1%	0.5 (0, 2)	2.5 (1, 3)	0.5 (0, 2)
	Lupeol 0.2%	1 (0, 3)	3 (2, 3)	0.5 (0, 1) *
	Lupeol 0.4%	1.5 (0, 3)	3 (2, 3) *	0.5 (0, 1) *
**Granulation**	Lanette	0 (0, 1)	1 (0, 2)	2 (1, 3)
	Collagenase	1 (0, 2)	2 (1, 3)	1 (1, 3)
	Lupeol 0.1%	1 (0, 2)	2 (1, 3)	1 (0, 2)
	Lupeol 0.2%	1 (0, 1)	2 (2, 3) *	1 (0, 2)
	Lupeol 0.4%	1 (0, 2)	2.5 (1, 3) *	1 (0, 2)

Data are expressed as median (minimum and maximum) and analyzed by the Kruskal–Wallis test, followed by the Dunn test. * *p* < 0.05 vs. Lanette group. (–) means parameter corresponding to the absence of observed signals.

**Table 2:** Sequence of primers used in RT-qPCR.

Gene	Primers Sequence 5′-3′	Product Size	Melting Temperature	Access Number *
*β-actin*	FW: CCCTGGCTCCTAGCACCAT RV: GATAGAGCCACCAATCCACACA	80 pb	60 °C	NM_031144.3
*Nf-κb*	FW: CCTCATCTTTCCCTCAGAGCC RV: CGCACTTGTAACGGAAACGC	98 pb	60 °C	NM_199267.2
*Ki-67*	FW: GGGTTTCCAGACACCAGACC RV: CCAGGAAGACCAGTTAGAACC	100 pb	60 °C	NM_001271366.1
*Egf*	FW: CTCAGGCCTCTGACTCCGAA RV: ATGCCGACGAGTCTGAGTTG	93 pb	60 °C	NM_012842.1
*Vegf-A*	FW: TGCGGATCAAACCTCACCAA RV: GGCTCACAGTGATTTTCTGGC	115 pb	60 °C	NM_001110333.2
*Tgf-β1*	FW: GGGCTACCATGCCAACTTCTG RV: GAGGGCAAGGACCTTGCTGTA	82 bp	60 °C	NM_021578.2

FW (forward), RV (reverse), and pb (base pairs). * National Center for Biotechnology Information, Nucleotide (NCBI-https://www.ncbi.nlm.nih.gov).

## Supplementary Materials

Supplementary materials can be found at https://www.mdpi.com/1422-0067/21/14/4952/s1.

## Abbreviations

Akt	Protein kinase B
ANOVA	One-way analysis of variance
cDNA	Complementary DNA
Cq	Quantification cycle
Ct	Cycle threshold
ddCT	Overall average of cycle threshold
ERK	Extracellular-signal-regulated kinase
EGF	Epidermal *growth factor*
ELISA	Enzyme-linked immunosorbent assay
EtOHE	Ethanolic extract
FGF-2	Fibroblast *growth factor-2*
FW	Forward
HE	Hematoxylin and eosin
IL-10	Interleukin 10
IL-1β	Interleukin-1beta
IL-6	Interleukin 6
Ki-67	Antigen Ki-67
MAPK	Mitogen-activated protein kinase kinase kinases
mRNA	Messenger RNA
NCBI	National Center for Biotechnology Information
NF-κB	Factor nuclear kappa B
PCR	Polymerase *chain* reaction
PDGF	Platelet-derived growth factor
PI3K	Phosphatidylinositol-3-kinase
RT-qPCR	Quantitative *reverse transcription PCR*
RV	Reverse
SC	Subcutaneous
SEM	Standard error of the mean
TGF-β1	*Transforming growth factor-beta* 1
TNF-α	Tumor necrosis factor alpha
VEGF	Vascular endothelial *growth factor*
