# Supplementary material for: From Inflammation to Cutaneous Repair: Topical Application of Lupeol Improves Skin Wound Healing in Rats by Modulating the Cytokine Levels, NF-κB, Ki-67, Growth Factor Expression, and Distribution of Collagen Fibers
Source: Int J Mol Sci. 2020 Jul 13;21(14):4952. doi: 10.3390/ijms21144952 (PMC7404060; doi:10.3390/ijms21144952)
Supplement: Supplementary file 1 [file ijms-21-04952-s001.pdf]

Supplementary Materials - Photomicrography of the NFκB, Ki-67, VEGF and EGF immunostaining in the border and lesion center of rats topically treated with lanette, collagenase 1.2 U/g or lupeol 0.2% creams

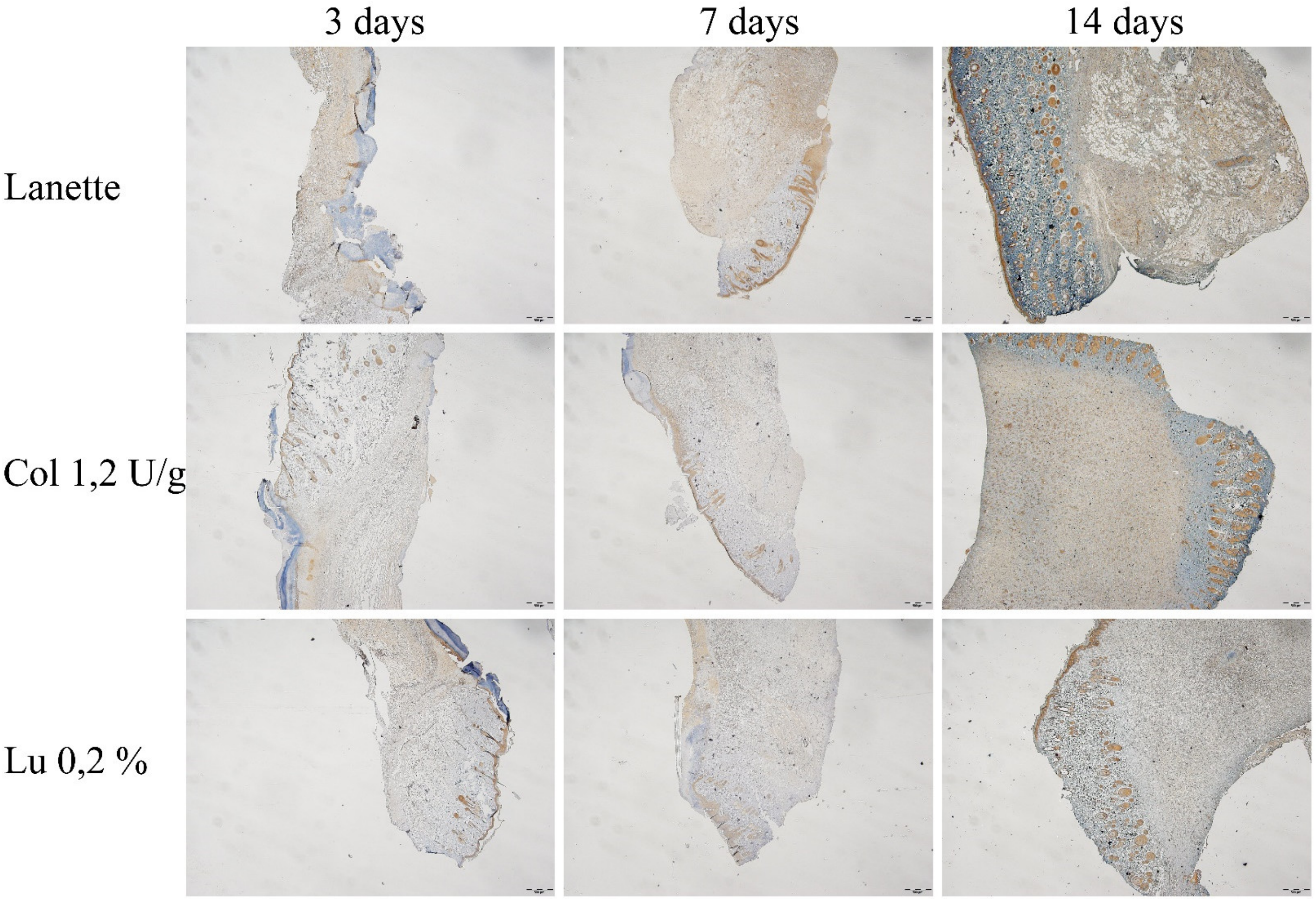

Supplementary Fig. S1: Photomicrography of the NFκB immunostaining in skin tissue sections on day 3, 7 and 14 post wound induction in low magnification. Bar represents 500μm.

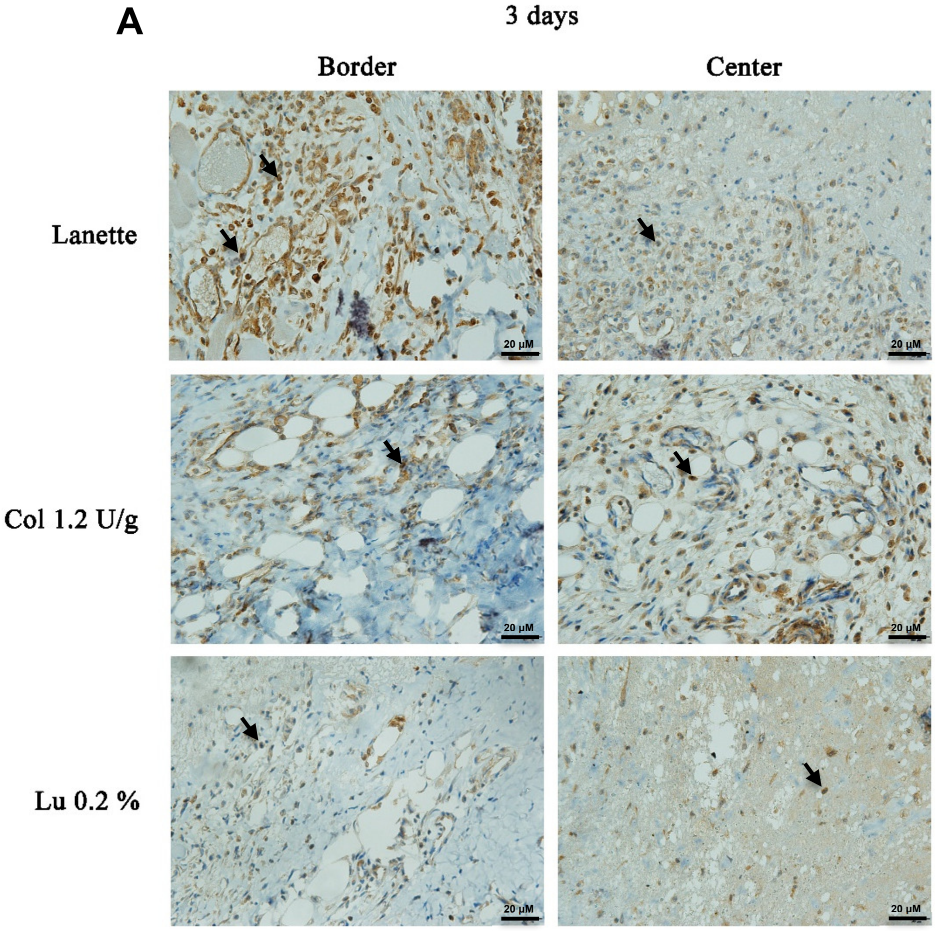
A
3 days
Border
Center
Lanette
Col 1.2 U/g
Lu 0.2 %
20 μM

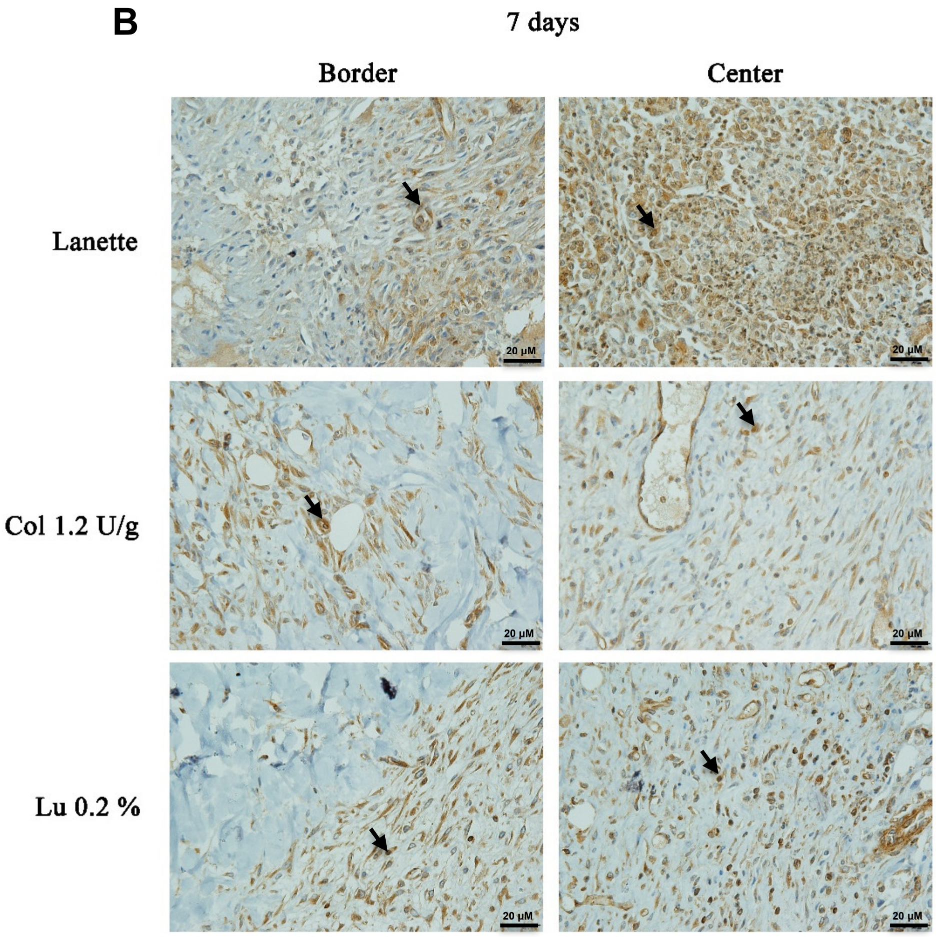
B
7 days
Border
Center
Lanette
Col 1.2 U/g
Lu 0.2 %
20 µM

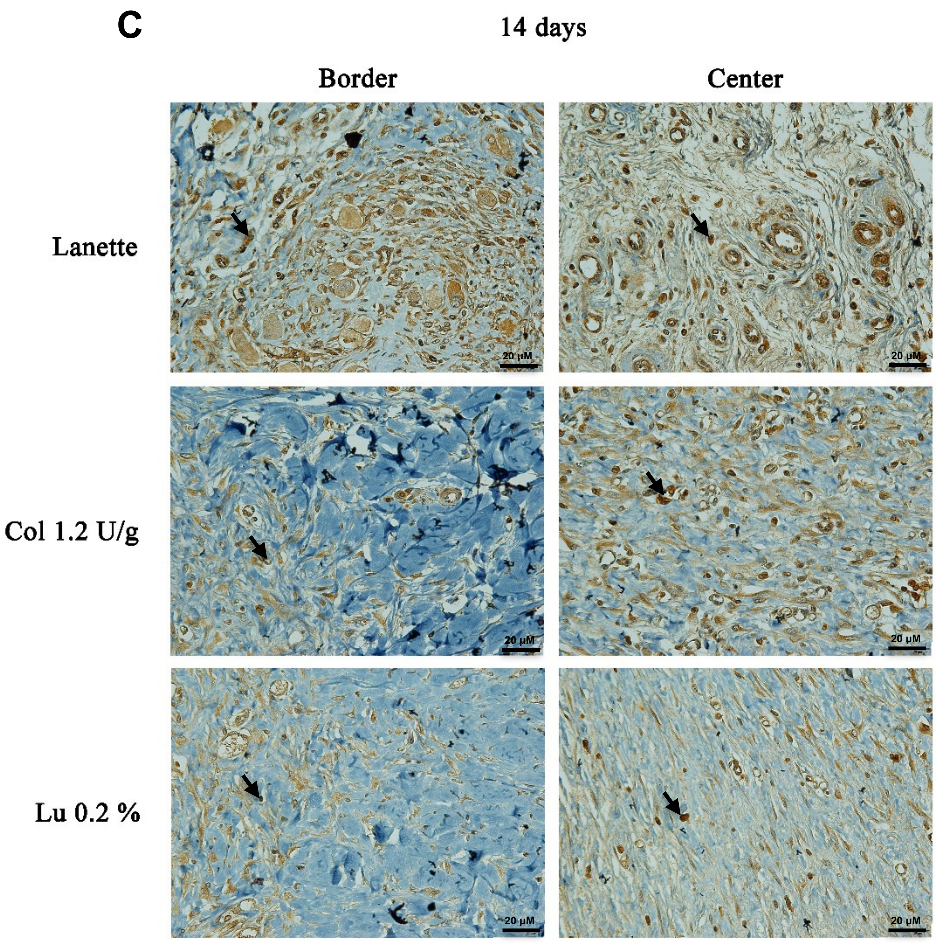

Supplementary Fig. S2: Photomicrography of the NFκB immunostaining in the border and lesion center of rats topically treated with lanette, collagenase 1.2 U/g or lupeol 0.2% creams in high magnification. A: 3 days after wound induction. B: 7 days after wound induction. C: 14 days after wound induction. Bar represents 20μm. Col: collagenase. Lu: lupeol. Black arrows indicate antibody staining against NF-κB.

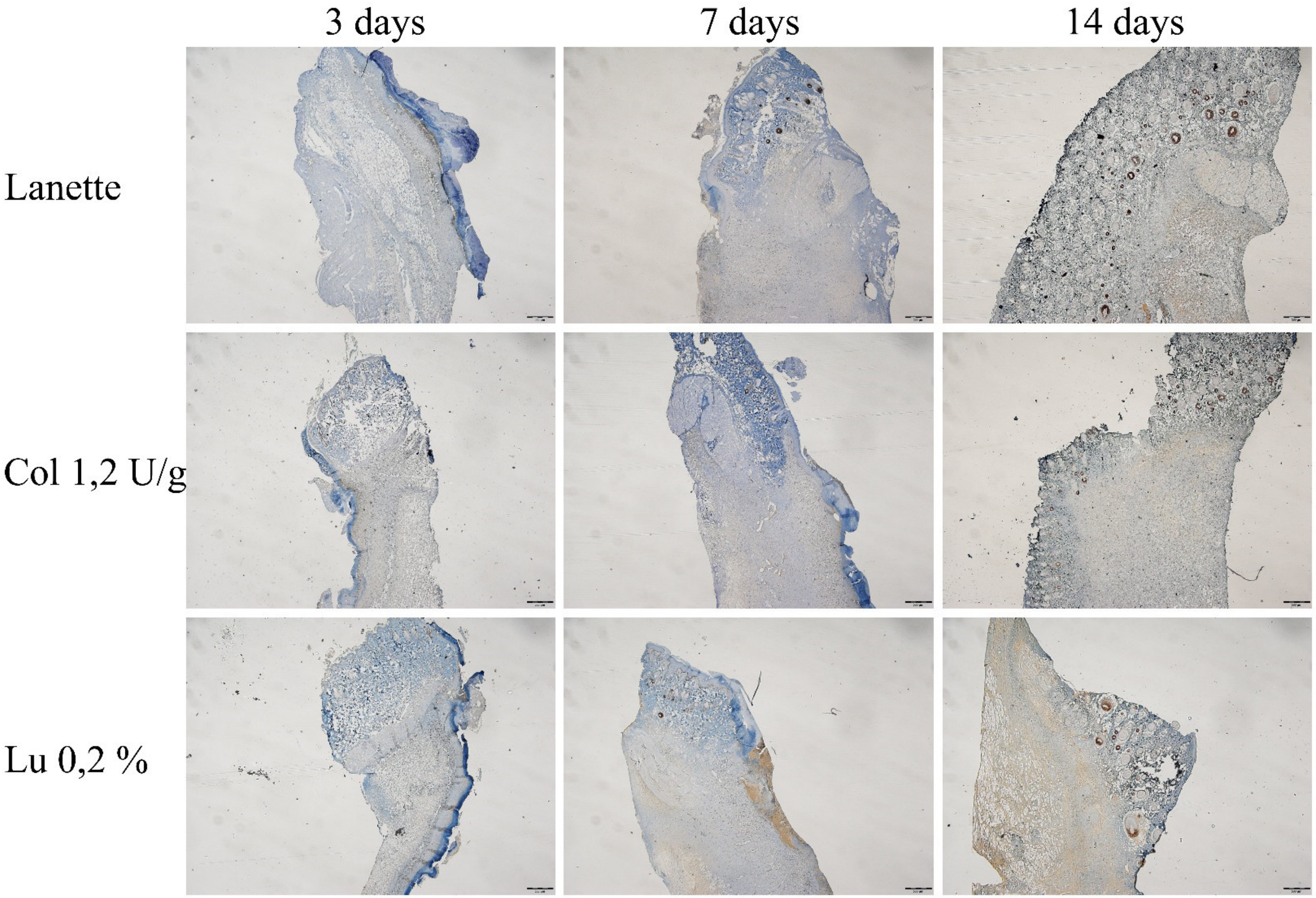

Supplementary Fig. S3: Photomicrography of the Ki-67 immunostaining in skin tissue sections on day 3, 7 and 14 post wound induction in low magnification. Bar represents 500µm.

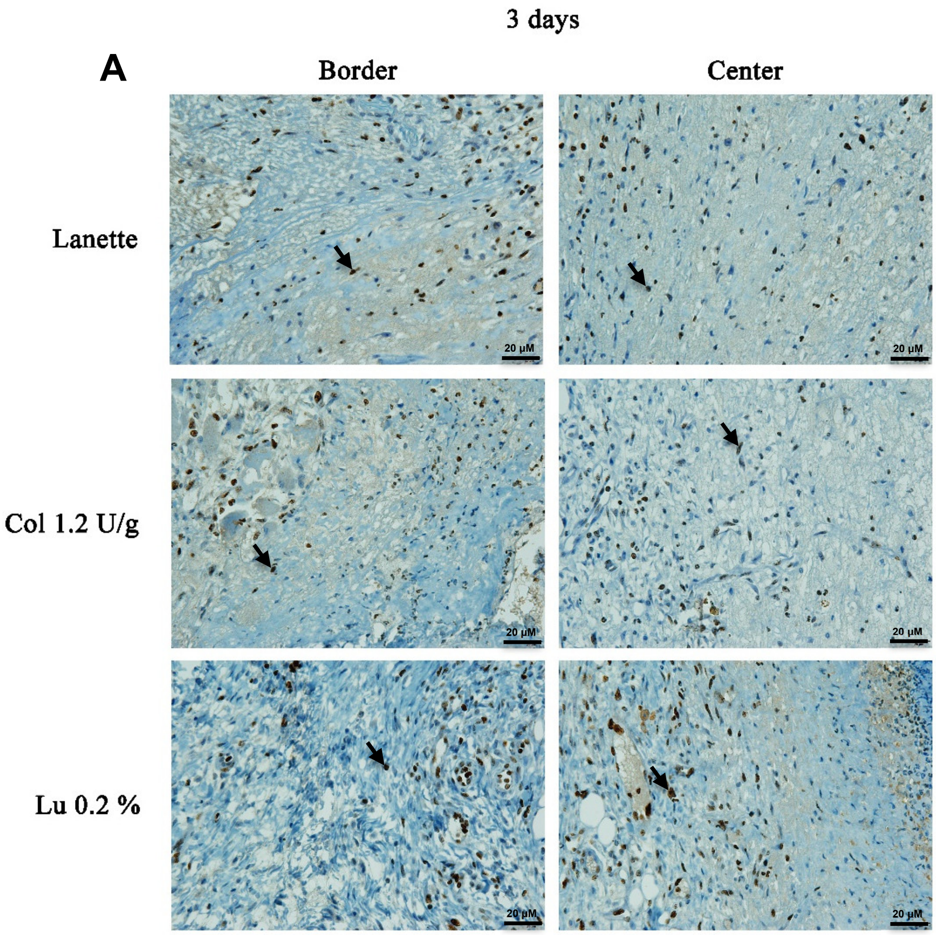
3 days
A
Border
Center
Lanette
Col 1.2 U/g
Lu 0.2 %
20 µM

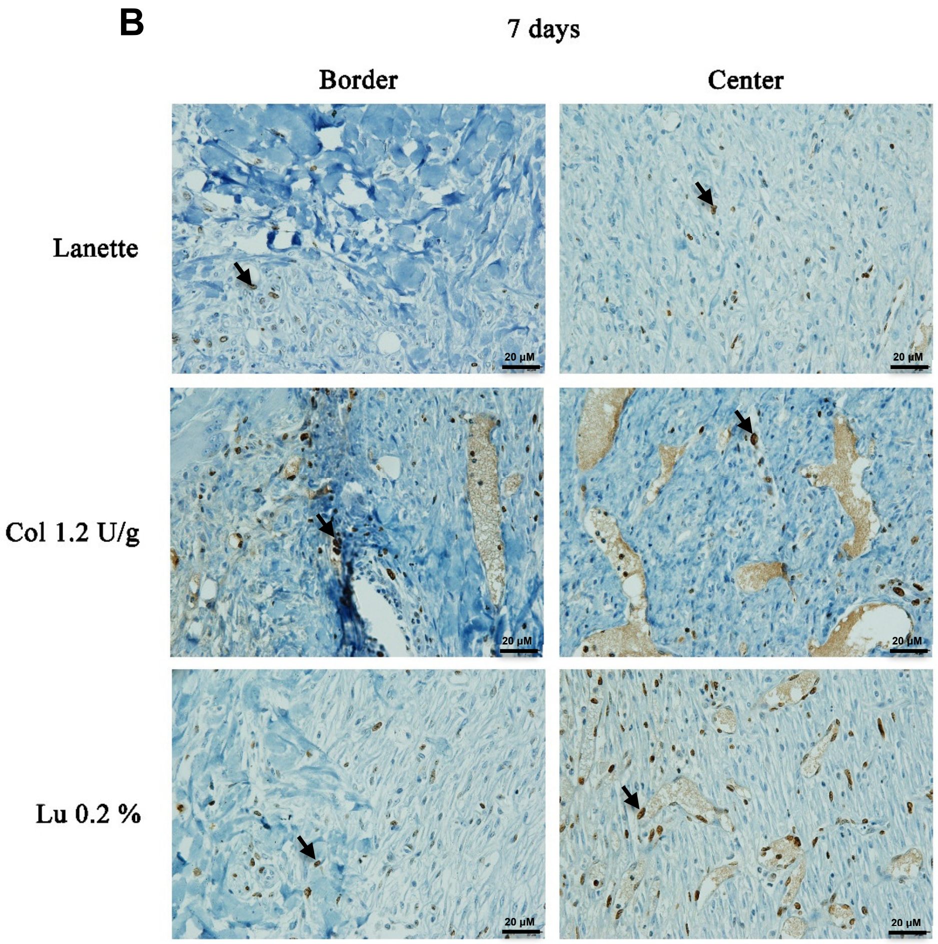
B
7 days
Border
Center
Lanette
Col 1.2 U/g
Lu 0.2 %
20 μM

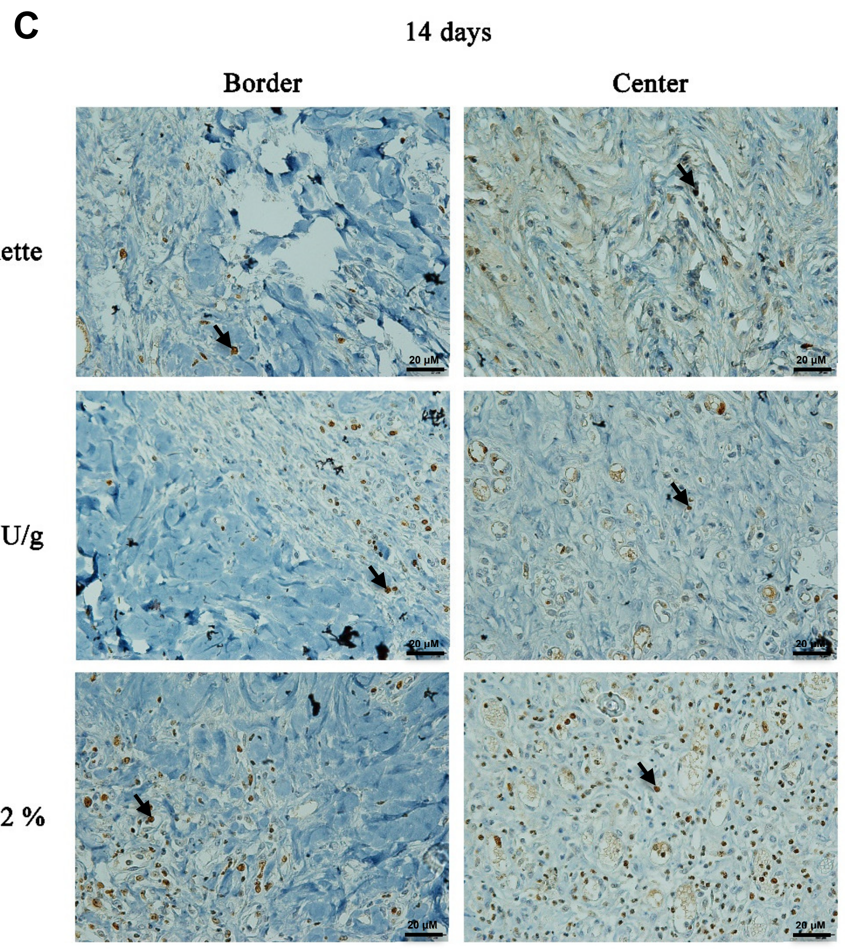

Supplementary Fig. S4: Photomicrography of the Ki-67 immunostaining in the border and lesion center of rats topically treated with lanette, collagenase 1.2 U/g or lupeol 0.2% creams in high magnification. A: 3 days after wound induction. B: 7 days after wound induction. C: 14 days after wound induction. Bar represents 20μm. Col: collagenase. Lu: lupeol. Black arrows indicate antibody staining against Ki-67.

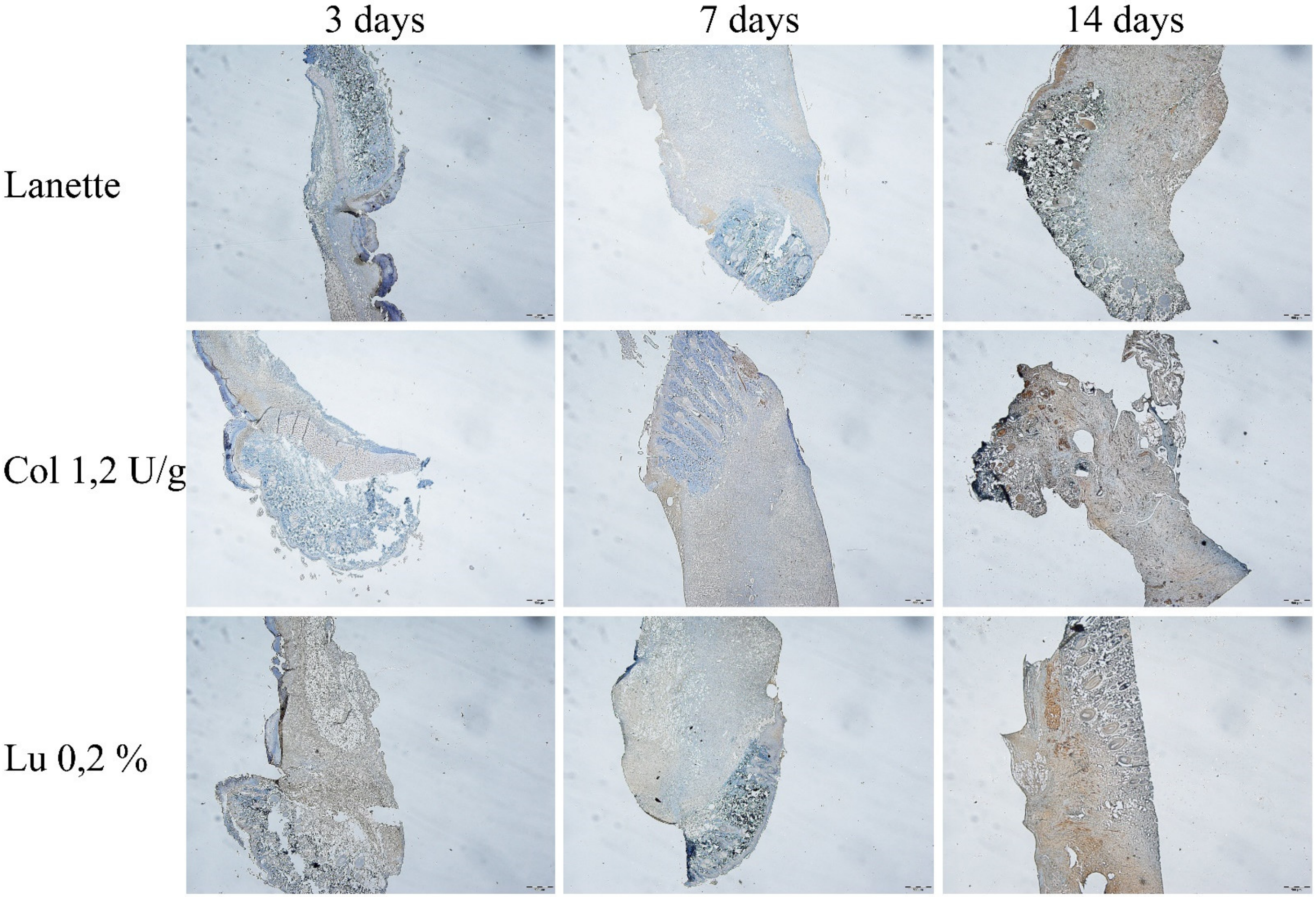

Supplementary Fig. S5: Photomicrography of the VEGF immunostaining in skin tissue sections on day 3, 7 and 14 post wound induction in low magnification. Bar represents 500µm.

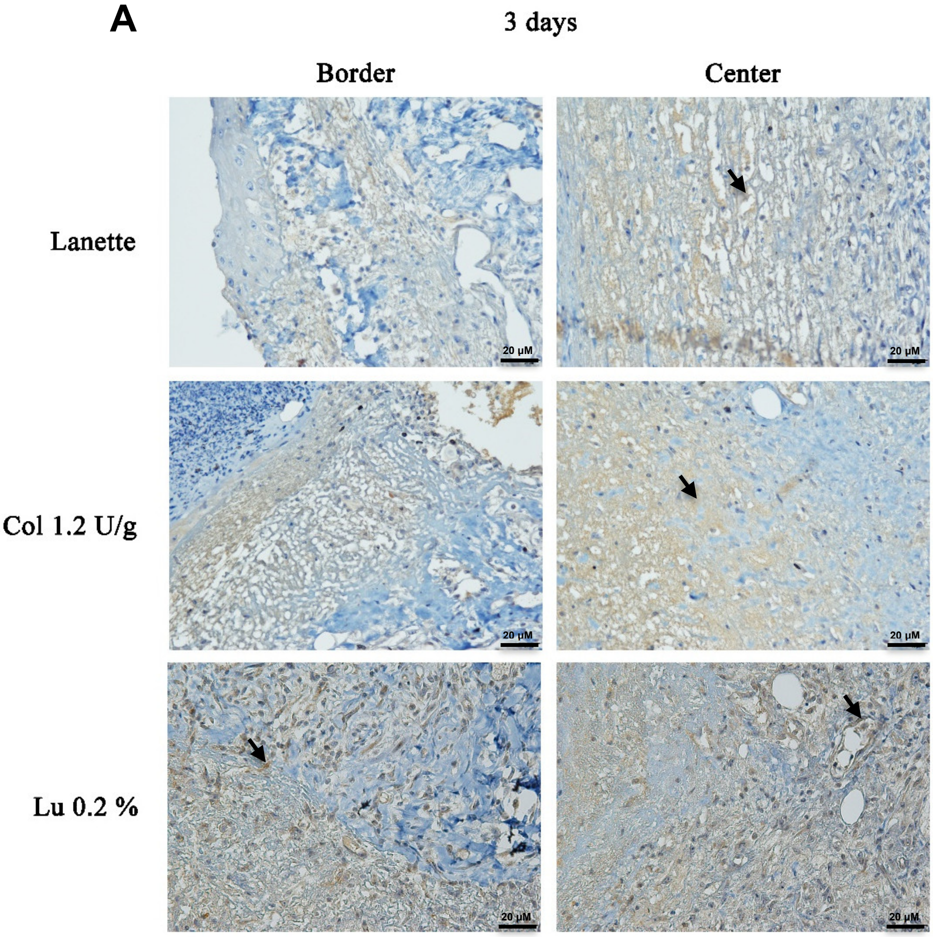
A
3 days
Border
Center
Lanette
Col 1.2 U/g
Lu 0.2 %
20 μM

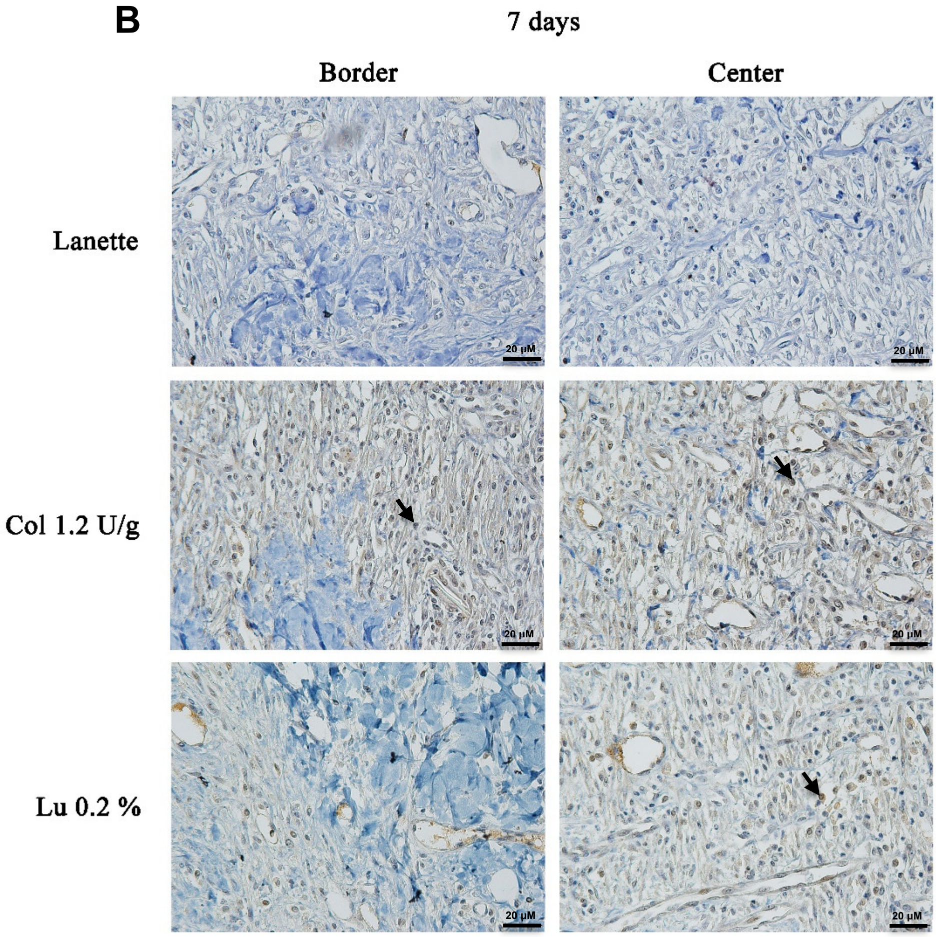
B
7 days
Border
Center
Lanette
Col 1.2 U/g
Lu 0.2 %
20 μM

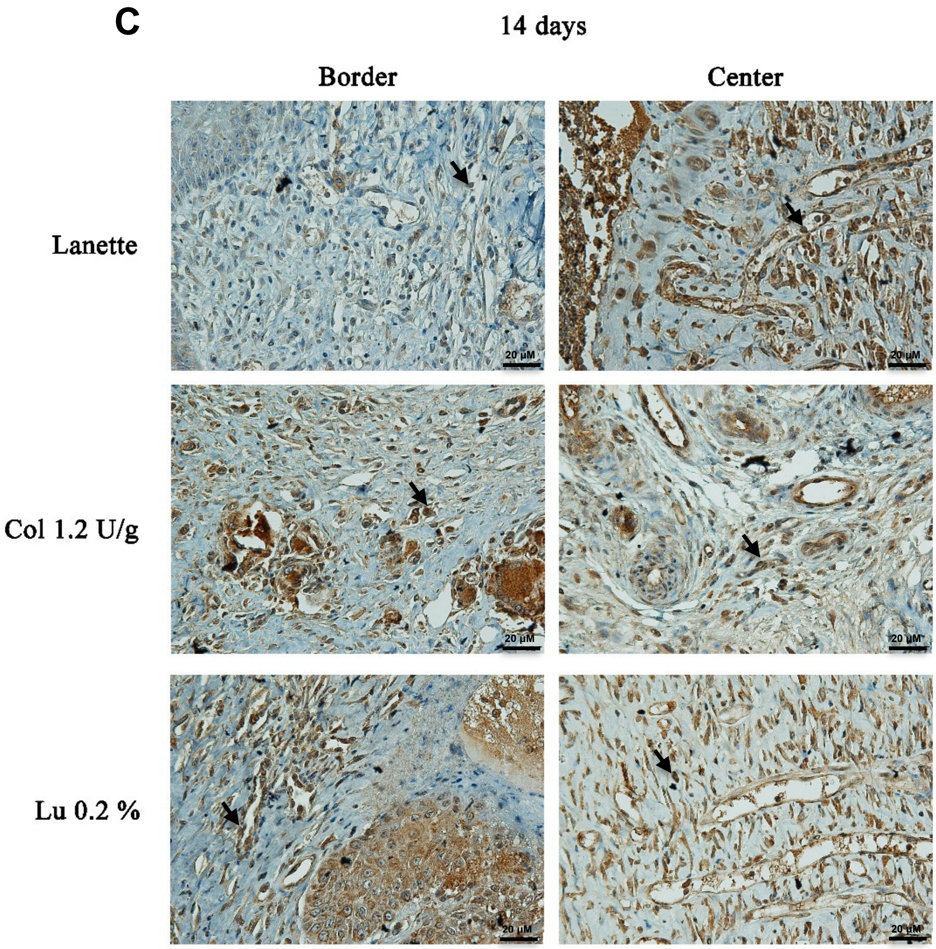

Supplementary Fig. S6: Photomicrography of the VEGF immunostaining in the border and lesion center of rats topically treated with lanette, collagenase 1.2 U/g or lupeol 0.2% creams in high magnification. A: 3 days after wound induction. B: 7 days after wound induction. C: 14 days after wound induction. Bar represents 20µm. Col: collagenase. Lu: lupeol. Black arrows indicate antibody staining against VEGF.

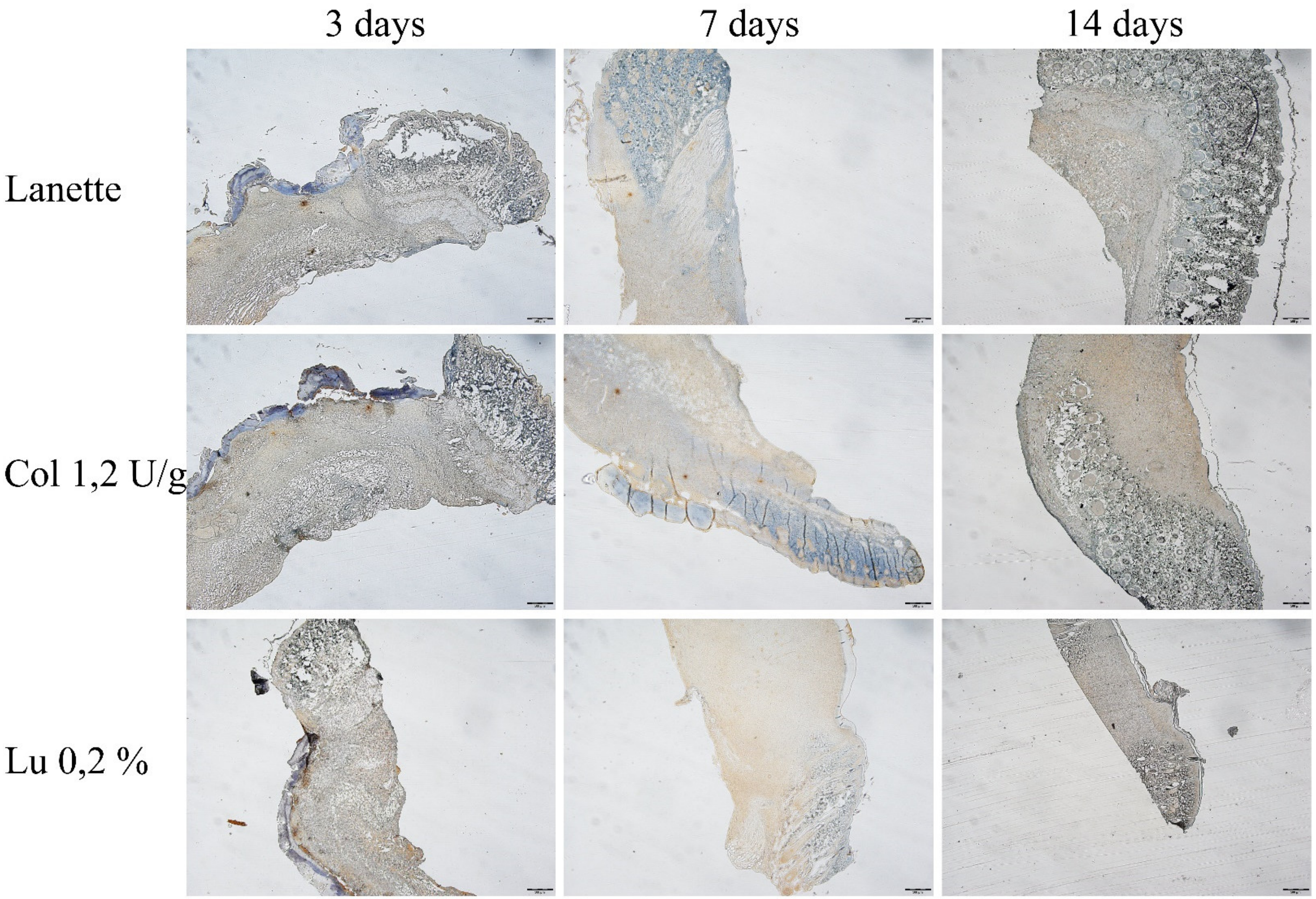

Supplementary Fig. S7: Photomicrography of the EGF immunostaining in skin tissue sections on day 3, 7 and 14 post wound induction in low magnification. Bar represents 500µm.

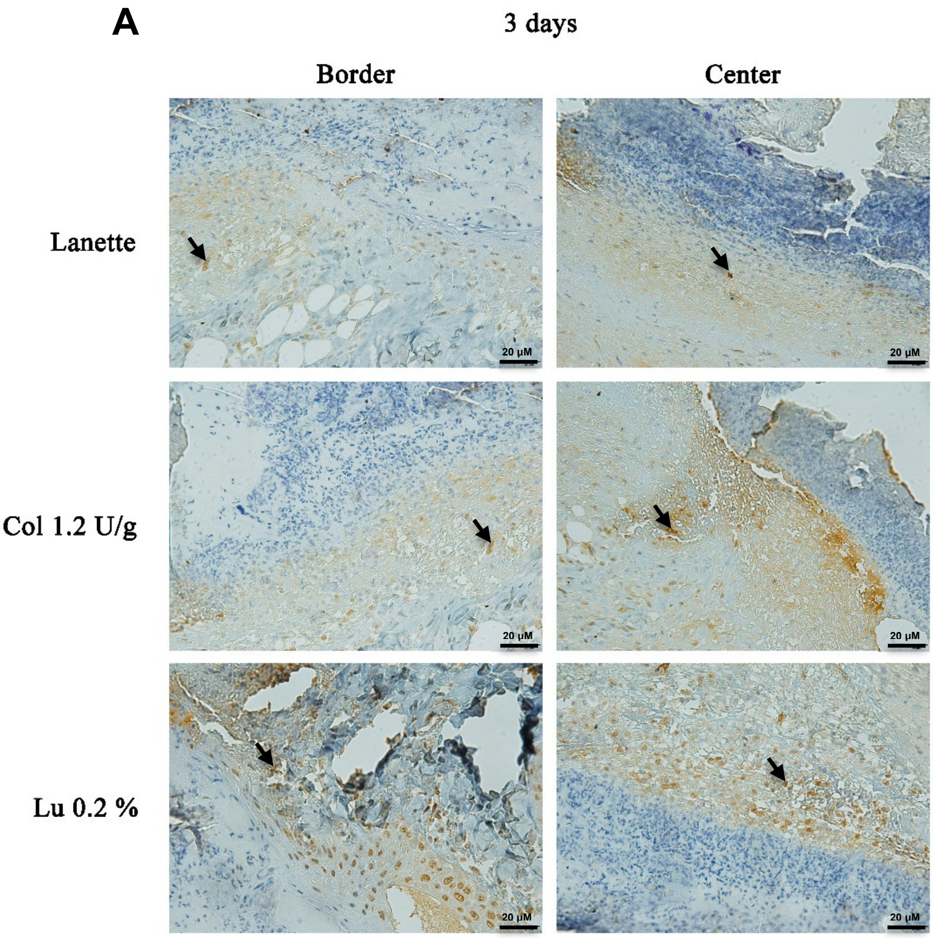
A
3 days
Border
Center
Lanette
Col 1.2 U/g
Lu 0.2 %
20 μM

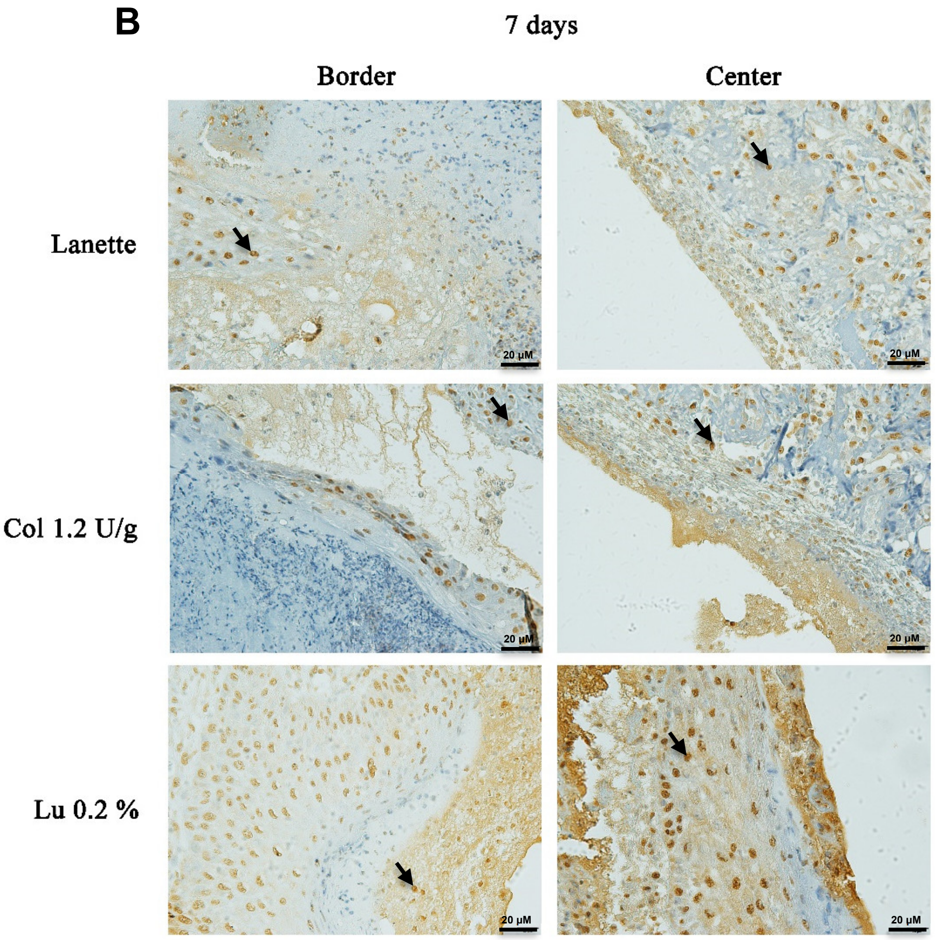
B
7 days
Border
Center
Lanette
Col 1.2 U/g
Lu 0.2 %
20 μM

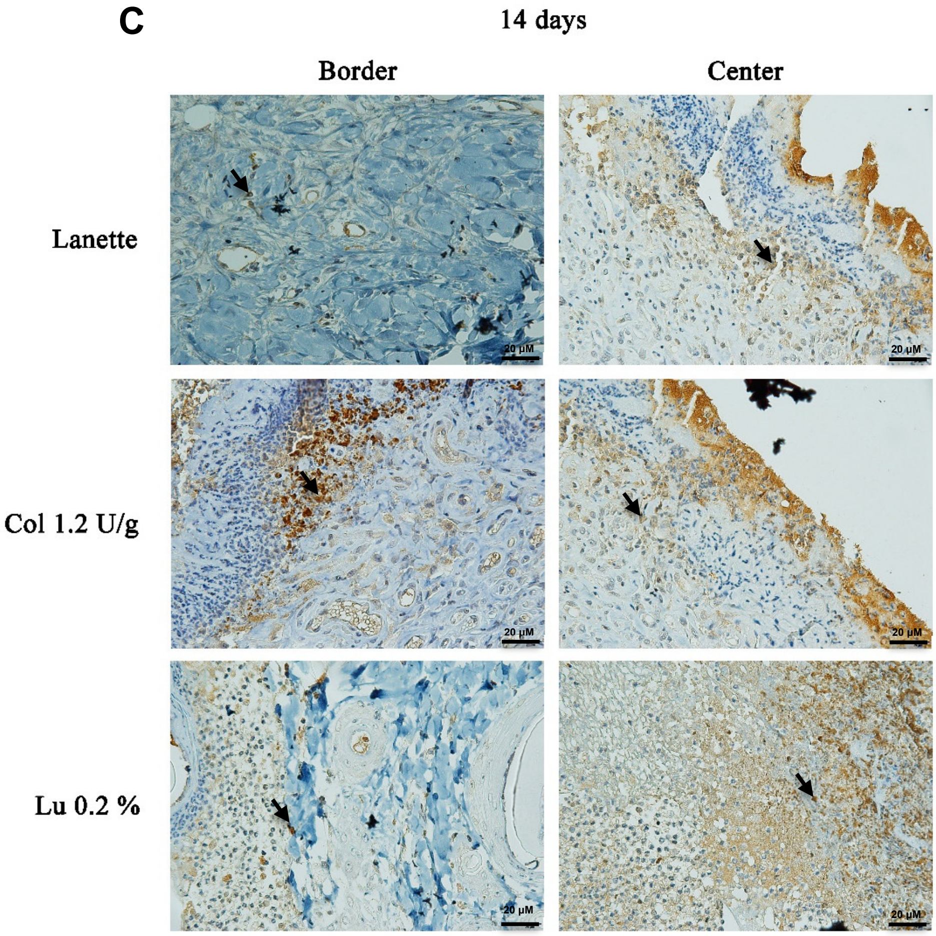

Supplementary Fig. S8: Photomicrography of the EGF immunostaining in the border and lesion center of rats topically treated with lanette, collagenase 1.2 U/g or lupeol 0.2% creams in high magnification. A: 3 days after wound induction. B: 7 days after wound induction. C: 14 days after wound induction. Bar represents 20µm. Col: collagenase. Lu: lupeol. Black arrows indicate antibody staining against EGF.
